# Regression Model-Based Walking Speed Estimation Using Wrist-Worn Inertial Sensor

**DOI:** 10.1371/journal.pone.0165211

**Published:** 2016-10-20

**Authors:** Shaghayegh Zihajehzadeh, Edward J. Park

**Affiliations:** School of Mechatronic Systems Engineering, Simon Fraser University, 250–13450 102^nd^ Avenue, Surrey, BC, V3T 0A3, Canada; West Virginia University, UNITED STATES

## Abstract

Walking speed is widely used to study human health status. Wearable inertial measurement units (IMU) are promising tools for the ambulatory measurement of walking speed. Among wearable inertial sensors, the ones worn on the wrist, such as a watch or band, have relatively higher potential to be easily incorporated into daily lifestyle. Using the arm swing motion in walking, this paper proposes a regression model-based method for longitudinal walking speed estimation using a wrist-worn IMU. A novel kinematic variable is proposed, which finds the wrist acceleration in the principal axis (i.e. the direction of the arm swing). This variable (called *pca-acc*) is obtained by applying sensor fusion on IMU data to find the orientation followed by the use of principal component analysis. An experimental evaluation was performed on 15 healthy young subjects during free walking trials. The experimental results show that the use of the proposed *pca-acc* variable can significantly improve the walking speed estimation accuracy when compared to the use of raw acceleration information (*p*<0.01). When Gaussian process regression is used, the resulting walking speed estimation accuracy and precision is about 5.9% and 4.7%, respectively.

## Introduction

Walking speed is widely used to study human health status. Based on previous studies, walking speed can be used as a marker of mild cognitive impairment (MCI) [1–3]. For example, the trajectories of weekly walking speed and the coefficient of variation of the walking speed are shown to be among the most important parameters for the early detection of MCI in older adults [1]. In addition to MCI, walking speed can also be used as a marker of multiple sclerosis (MS) [4], Parkinson’s disease [5, 6], risk of falls [7], kidney disease [8], and adverse outcomes in aging [9]. Hence, it can be considered as a powerful predictor of hospitalization, disability, and survival [10, 11]. In a clinical setting, different protocols including the 4-meter [12], 10-meter [8], and 6-minute walking tests [13] and the timed up and go (TUG) test [9, 14] have been used as standard tools to evaluate walking speed and gait parameters. However, the short walking tests (e.g. the 10-meter walking test) are subject to bias due to their brevity [15] and the longer tests are less accepted due to the space and time constraints in clinical exams [16]. Additionally, the walking speed results of clinical tests cannot be fully applied to the free-living environment [17]. Furthermore, building precise pathological models of some disease for development of monitoring and treatment guidelines, requires access to longitudinal measurements [18,19]. This emphasizes the need for a reliable system/method for longitudinal (i.e. over long periods of time) and continuous walking speed measurement in real-world situations.

Aiming at longitudinal walking speed measurement outside the clinical setting, some researchers have used passive infrared (PIR) motion sensors. These PIR sensors can be mounted on ceiling [20] or walls [21] of a residence and can measure the individuals’ walking speed when they are in the field of view of the sensors. However, walking speed measurement based on PIR sensors is limited to confined areas such as hallways. Additionally, such system cannot differentiate between multiple residents, limiting its application to independent-living resident homes. Camera-based systems have also been used in the literature for in-home gait speed measurement [22]. However, camera-based systems can get affected by the lighting conditions, and similar to the PIR sensors, they are limited to confined areas and hence more suitable for clinical settings.

Fortunately, with recent advances in MEMS technology and wireless sensor networks, wearable inertial measurement units (IMU) have emerged as powerful devices for portable human motion analysis [23–29]. Being self-contained, wearable inertial sensors can facilitate walking speed measurement in an ambulatory fashion. Considering that the acceleration data from tri-axial accelerometer in a wearable inertial sensor can be integrated to get the velocity, integration-based approaches have been widely used for speed tracking [30]. The main challenge in integration-based approaches is the velocity drift over time that happens as a result of time-varying bias in MEMS-based inertial sensors [31]. To mitigate the drift, some researchers have proposed the detection of periodic foot stance phases during walking to reset the velocity to zero through a process called zero velocity update (ZUPT) [30–34]. However, the need for foot-stance detection requires the wearable sensor to be normally mounted on the leg (ideally on the foot), which is inconvenient for longitudinal walking speed monitoring, particularly indoors. Using waist-worn IMU, some studies have modeled the foot swing in walking as an inverted pendulum to find a 3D walking kinematic model for speed estimation [35]. Additionally, using a waist-mounted IMU, linear and nonlinear regression models have shown promising performances for ambulatory walking [13, 36–37] and swimming [38] speed estimation. These regression-based approaches for walking speed estimation are based on mapping the inherent pattern of acceleration and rate of turn information corresponding to the hip rotation in a gait cycle to walking speed.

For longitudinal health status monitoring, among the available state-of-the-art inertial sensing-based wearables, wrist-worn devices are the most user-friendly and compliant that do not limit the freedom of movement and do not require specific dressing style (e.g. wearing a belt in the case of waist-worn sensor). Thus, wrist-worn devices have relatively higher potential to be easily incorporated into daily lifestyle and worn for longer hours. Similar to hip rotation in each gait cycle [13], arm swing motion during walking is a periodic motion pattern that is highly correlated to walking speed: the faster the walking speed, the faster the arm swing motion. However, in walking speed estimation based on regression models, free arm motion necessitates the use of more complex algorithms to manipulate the acceleration and rate of turn information and get a variable that is more representative of the arm swing motion. Although extracting this variable is of high importance (because the accuracy of the regression models depends on the set of chosen variables and the extracted features), it has not been addressed in the existing literature.

Aiming at accurate walking speed estimation using a wrist-worn IMU, this paper provides a novel processing method based on combined inertial sensor fusion and principal component analysis (PCA) for variable extraction. Experimental results show that the extracted variable can improve the accuracy of wrist-based walking speed estimation.

## Theoretical Method

### Problem Definition

Considering that walking is represented by a set of features, this section is focused on formulating a mapping from walking-related features (predictors) to walking speed (response value) using a regression model. In a regression problem, a training set (S) consisting of *N*-number of *D*-dimensional predictors ***x***_*i*_ and noisy observations of the response value *y*_*i*_ is given ($S={\left\{\left(x_{i},y_{i}\right)\right\}}_{i=1}^{N}$). The goal of a regression model is to find the best-fit function *f*(***x***_*i*_) that predicts the response values. The Gaussian process regression (GPR) and regularized least squares regression using least absolute shrinkage and selection operator (LSR-Lasso) models are the two candidate regression methods used in this study.

### Gaussian Process Regression

The objective of GPR, a well-known non-parametric regression technique, is to model the dependency as follows [39]:

$$y_{i}=\;f\left(x_{i}\right)+\varepsilon_{i}$$ (1)

where *ε*_*i*_ = *N*(0, *σ*_*n*_^2^) is the independent and identically, normally distributed noise terms. GPR has two main advantages compared to conventional regression methods [40]:

1. It is a non-parametric regression method and the model structure is determined from data.
2. It uses a probabilistic approach that can model the prediction uncertainty.

A Gaussian process is completely identified by its mean *μ*(***x***_*i*_) and covariance function ***Σ***(*x*_*i*_, *x*_*j*_). The covariance function used here is a parameterized squared exponential (SE) covariance function [39]:

$$\Sigma \left(x_{i},x_{j}\right)=\sigma_{f}{}^{2}\text{exp}\left(-\frac{1}{2\left(x_{i}-x_{j}\right)W^{-1}{\left(x_{i}-x_{j}\right)}^{T}}\right)$$ (2)

where *σ*_*f*_ is the signal variance and ***W*** = *diag*(*l*_1_, …, *l*_*D*_) is the diagonal matrix of length-scale parameters. This covariance function implements automatic relevance determination (ARD) as the length-scale values determine the effect of each predictor on the regression.

Assuming *N* training samples are available, for a new input, x*, the covariance matrix in Eq (2) can be partitioned into two blocks [39]:

$$\Sigma_{N+1}=\left(\begin{array}{cc} \Sigma_{N} & \Sigma_{N^{*}}\\ \Sigma_{N^{*}}^{T} & \alpha \end{array}\right)$$ (3)

where *Σ*_*N*_ is the *N* × *N* covariance matrix of the training samples, $\Sigma_{N^{*}}$ includes the elements of ***Σ***(***x***_*i*_, x*) and *α* represents ***Σ***(x*, x*). A posteriori mean estimation and related variance can be given respectively by

$$\mu \left(x^{*}\right)=\Sigma_{N^{*}}\;\Sigma_{N}^{-1}\;y$$ (4)

$$\sigma^{2}\left(x^{*}\right)=\alpha -\Sigma_{N^{*}}^{T}\;\Sigma_{N}^{-1}\;\Sigma_{N^{*}}$$ (5)

where ***y*** = [*y*_1_, …, *y*_*N*_]^*T*^.

GPR is chosen herein because of its superior performance compared to other regression models in [36] where a waist-worn IMU is used to estimate walking speed.

### Regularized Least Squares Regression Using Lasso

Lasso is the shrinkage and selection method for regularized linear regression. LSR-Lasso, a well-known parametric regression technique, minimizes the usual sum of squared errors, with a bound on the sum of the absolute values of the coefficients to deliver a sparse solution, i.e. a set of estimated regression coefficients in which only a small number is non-zero [41]. Given a linear regression, the LSR-Lasso solves the ℓ_1_-penalized regression to minimize [41]:

$$\frac{1}{2}\Sigma_{i=1}^{N}{\left(y_{i}-\beta_{0}-x_{i}{}^{T}\beta \right)}^{2}+\lambda \Sigma_{j=1}^{D}\left|\beta_{j}\right|$$ (6)

for unknown parameters *β*_0_ and ***β*** = [*β*_1_, …, *β*_*D*_]. The second term in Eq (6) is the penalty function balancing the fit of the model with its complexity with the non-negative parameter *λ* governing this trade-off [41]. The value of *λ* is chosen based on 10-fold cross-validation in this study.

LSR-Lasso is chosen in this study to provide a performance baseline for GPR with the SE-ARD covariance function.

## Experimental Method

### Participants

Fifteen young (nine males, six females) self-reported healthy students from Simon Fraser University participated in this study. The participants had an average age of 27±4 years, average height of 1.69±0.08 m, average weight of 6510±10 kg, and average body mass index (BMI) of 23.07±2.3 kg/m^2^. Informed written consent was obtained from the participants and the experimental protocol (No. 2013s0750) was approved by the Office of Research Ethics of Simon Fraser University.

### Hardware and Experimental Protocol

**Fig 1:**
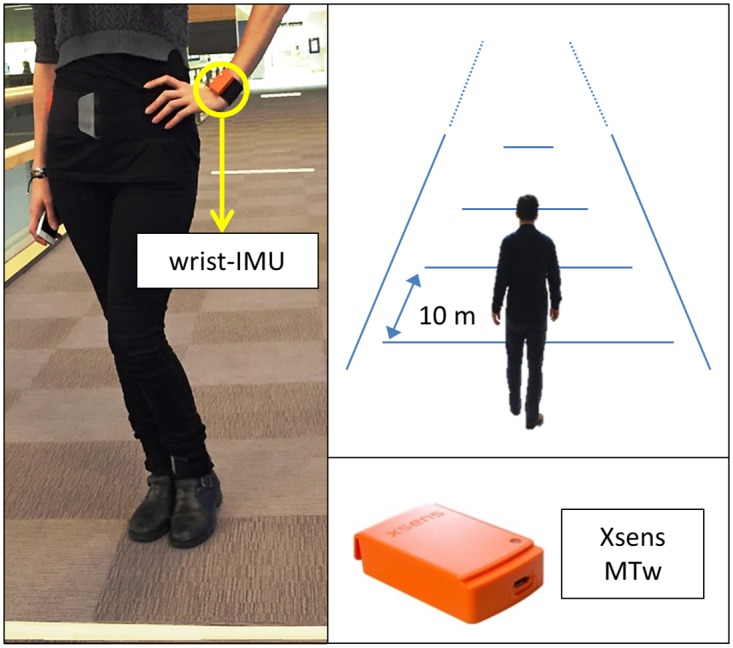
Experimental set-up. Left: a subject wearing MTw units; right: MTw unit and schematic of the test field.

Raw inertial and magnetic data are collected from tri-axial accelerometers, gyroscopes, and magnetometers at the rate of 100 Hz. The sensor is Xsens MTw worn by human subjects on the wrist (Fig 1). Each subject is asked to walk for a distance of 30 m in indoor environment for three different self-selected walking speed regimes: slow, normal, and fast. The subjects are asked to keep their walking speed constant during each 30 m trial and each trial is repeated four times per chosen speed regime, resulting in 12 trials per subject. To get the ground truth average walking speed, the floor is divided into three segments of 10 m long (accurately measured by a laser distance measuring tool with sub-centimeter accuracy), as shown in Fig 1, and the time it takes for the subject to pass each segment is measured using a stopwatch with an accuracy of 0.01 s. The criterion of line passage is when the subject’s right foot passes the line and a human observer always walked with the subject to ensure a perfect sagittal plane view.

For the purpose of demonstrating and further evaluating the performance of the proposed walking speed estimation method in a real-world setting, five subjects (four males, one female) are asked to perform a 12-min outdoor walking trial that includes 2 min of fast walking, 4 min of normal walking, and 6 min of slow walking. In these outdoor trials, Xsens MTi-G-700 [consisting of tri-axial accelerometers, tri-axial gyroscopes, tri-axial magnetometers, and the Global Positioning System (GPS)] is worn by the subjects on their left wrist and the reference walking speed is obtained by GPS/IMU fusion using our existing Kalman filter-based fusion algorithm previously presented in [31]. Compared to the indoor trials, these outdoor trials cover longer walking distances and durations and the subjects have the freedom to change their walking direction.

## Analysis

### Variable Computation

The raw data are used to calculate three different variables: magnitude of 3D acceleration (*acc*), magnitude of external acceleration (*ext-acc*), and external acceleration in the principal axis (*pca-acc*). The idea here is to start from raw acceleration data and apply step-by-step increasingly more advanced signal processing techniques to process the raw inertial data to get a variable that is more representative of the arm swing during walking. The above-mentioned three variables are explained in the following:

#### acc

The norm (square root of the sum of squares) of acceleration components:

acc= |as|=asx2+asy2+asz2 (7)

where ^*s*^*a*_*i*_,*i* = *x*,*y*,*z* is the acceleration measured by each axis of the accelerometer in the sensor frame (*s*-frame: a coordinate frame attached to the sensor).

#### ext-acc

**Fig 2:**
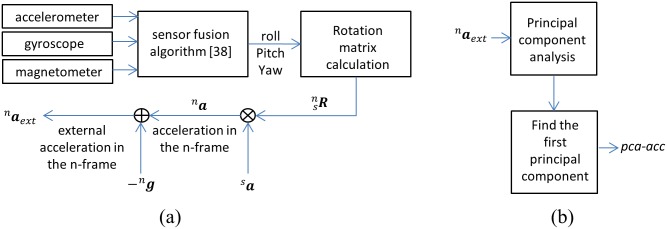
Variables calculation method. (a) *ext-acc* variable and (b) *pca-acc* variable.

This variable is the norm of gravity compensated acceleration (also known as external acceleration). Removing the gravity component from the tri-axial accelerometer data results in a variable that represents the pure acceleration of the arm during walking. The following steps are taken to get the *ext-acc* variable (Fig 2a):

- Orientation is obtained by fusing the tri-axial accelerometer, gyroscope, and magnetometer using our previous Kalman filter-based sensor fusion algorithm in [42–44].
- The rotation matrix (Rsn) [42] is calculated to represent the acceleration in the navigation frame (^*n*^***a***). The navigation frame (*n*-frame) is a local-level frame with its *x*- and *y*-axis in the horizontal plane and its *z*-axis aligned with the gravity vector).
- The gravity component of the acceleration is then removed:
  anext=Rsnas−gn (8)
  where ^*n*^***a***_*ext*_ and ^*n*^***g*** are the tri-axial external acceleration vector and the gravity vector, respectively, both in the *n*-frame.
- Finally, the *ext-acc* is calculated as
  ext−acc=|anext|=anext,x2+anext,y2+anext,z2 (9)

#### pca-acc

This variable is the horizontal external acceleration in the direction of its highest variations. Considering the problem of walking speed estimation based on arm swing motion, one of the main shortcomings of the above 3D external acceleration (^*n*^***a***_*ext*_) is its dependency on the direction of arm swing motion in the navigation frame. The first issue is the inter-subject variability of the arm swing angle (i.e. for the same walking speed, the direction of arm swing with respect to the forward direction of motion varies between individuals). The second issue is the intra-subject variability for different walking directions (i.e. for the same walking speed, any changes in the walking direction result in a change in the absolute direction of the arm swing in the navigation frame). In the above-mentioned two scenarios, variations in the direction of arm swing result in changes in the components of ^*n*^***a***_*ext*_. This will affect the magnitude of the *ext-acc*, which in the regression model will be interpreted as a change in the walking speed. However, for a constant speed, the direction of arm swing should not affect the estimation of walking speed ideally. Thus, the *pca-acc* is proposed in here as a direction-independent variable. To obtain the *pca-acc* (Fig 2b), PCA [45] is applied on the first two components (the horizontal components) of ^*n*^***a***_*ext*_ to find the direction of the highest acceleration variation in the horizontal plane, which is aligned with the direction of arm swing. The *pca-acc* variable is simply the acceleration in this direction (the first principal component).

### Feature Extraction

The sensor data from the IMU are low-pass filtered using a Butterworth filter with a cut-off frequency of 20 H_Z_ considering that activities of daily living (ADL) fall in the frequency range of 0.1 to 10 H_Z_ [40]. Each of the above-mentioned three variable streams is divided into 5-s epochs. The 5-s window is selected based on the window size proposed in [36] and that the periodicity of the signal should be captured in this snapshot. Within each epoch, the following time-domain (TD) and frequency-domain (FD) features are calculated.

#### TD features

Eight TD features are used in this study including the statistical features consisting of mean, standard deviation (SD), median, mode, and mean of absolute values plus other features such as the number of mean crossing, signal magnitude area ($\sum_{n=1}^{N}\left|x\left[n\right]\right|$), and energy ($\sum_{n=1}^{N}x^{2}\left[n\right]$).

#### FD features

A 512-point fast Fourier transform (FFT) is used within each epoch to obtain frequency information. The first 40 coefficients of the single-sided amplitude spectrum are used as FD features. These 40 coefficients correspond to the frequency range of less than 8 Hz. This frequency range is selected based on the inspection of the Fourier transforms from the 15 subjects.

### Training versus Testing

Two modeling approaches have been compared in this study: subject-specific model versus generalized model. Considering *N* subjects, to train and test the models, for each subject, 20% of the subject-specific data set (of the short indoor tests) was randomly sampled and partitioned into test data, the remaining fraction constituting training data. The subject-specific model for subject *n* was trained using subject *n*’s training data and was tested on subject *n*’s test data. The generalized model for subject *n* was trained using all data from the remaining subjects and predictions were made on subject *n*’s test data. For each model, the process was repeated for 10 times for different randomly sampled data and the results were averaged. The final reported error is the error averaged across all participants.

For the generalized models, along with TD and FD feature sets, two anthropomorphic parameters including height and weight of the subjects are included in the input features. It is shown that these two features can potentially improve the generalizability of the models [46].

## Results and Discussion

### Effect of the PCA on External Acceleration Signal

**Fig 3:**
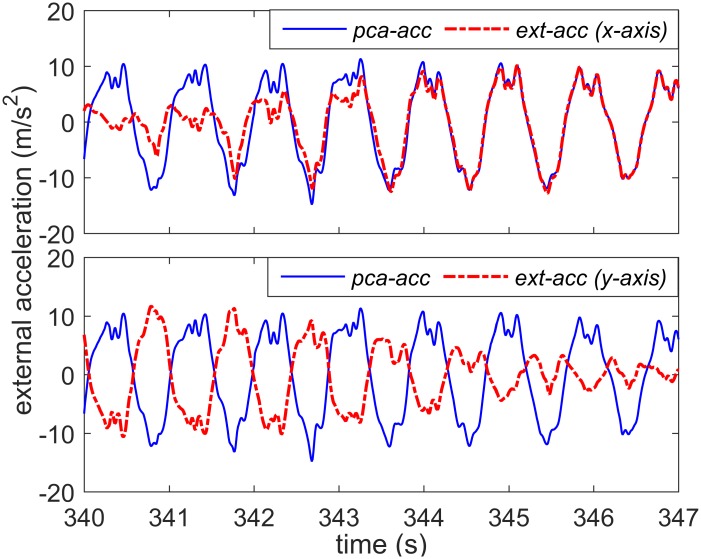
TD comparison between the variables. External acceleration signal in the directions of *x-* and *y*-axis of the *n*-frame and the principal axis (*pca-acc*) during 7 s of outdoor walking trial.

**Fig 4:**
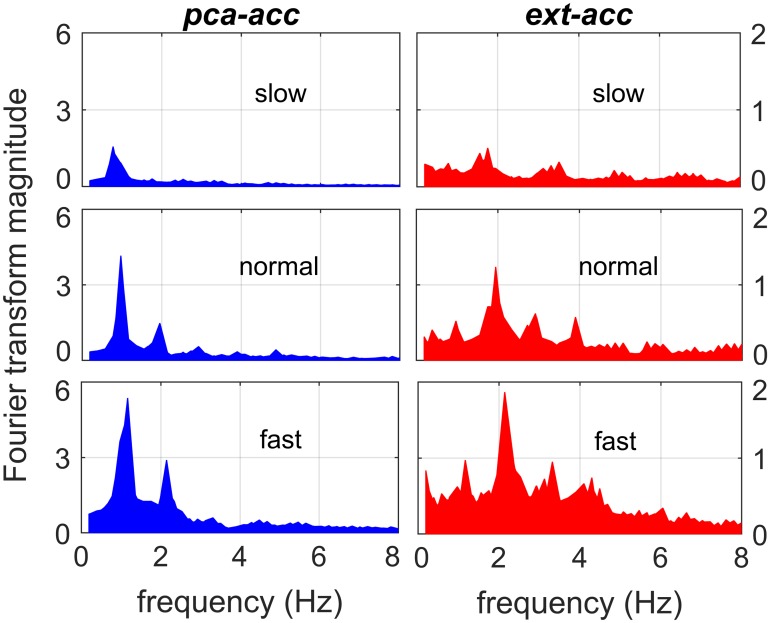
FD comparison between the variables. Fourier transform magnitude for the *pca-acc* and *ext-acc* signals from one subject.

Fig 3 shows the horizontal components of the external acceleration in the directions of *x*- and *y*-axis (of the *n*-frame) in addition to the direction of the principal axis (*pca-acc*) during 7 s of a 12-min outdoor walking trial. In this figure, the amplitude of the acceleration in the *x*-axis grows, whereas the one in the *y*-axis shrinks (happening when the walking direction changes). However, because the *pca-acc* signal always captures the acceleration in the principal axis (pointing toward the direction of motion), the amplitude of the *pca-acc* variable remains constant when the walking speed is constant, but the direction changes (see Fig 3). Thus, the *pca-acc* variable is expected to provide better estimates of walking speed compared to the external acceleration signal in each axis. Similar observation has also been made in [40]; when raw 3D acceleration data are used to estimate energy expenditure, the *x*-axis acceleration is coincidently aligned with the forward direction of motion, providing better accuracy compared to the *y*- and *z*-axis. On the contrary, the magnitude of 3D external acceleration (*ext-acc*) is another variable that will get affected less by the changes in walking direction compared to the acceleration in individual axes. In Fig 4, the *pca-acc* and *ext-acc* signals are compared to each other in the FD. This figure shows the Fourier transform magnitude for both *pca-acc* and *ext-acc* variables for the three different walking speed regimes (slow, normal, and fast) based on the data set collected from one subject. Based on this figure, compared to *ext-acc*, the *pca-acc* variable shares a relatively clearer pattern of peaks between the three walking regimes with the corresponding peaks moving to the higher frequencies and growing in amplitude as the walking speed gets faster. This clear pattern has the potential to provide relatively more accurate estimation of the walking speed.

### Performance of the Generalized GPR Model

**Fig 5:**
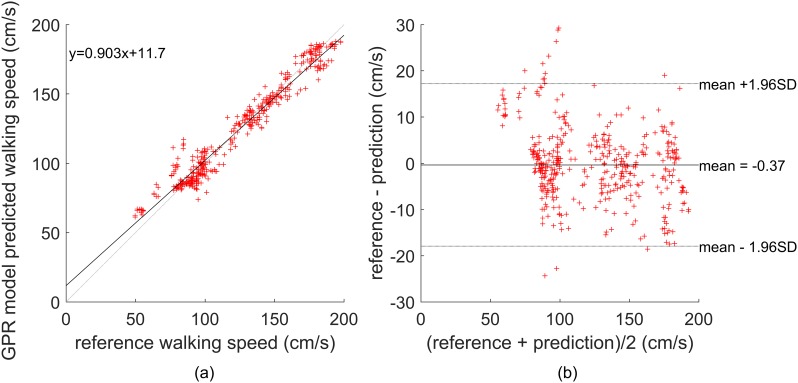
Regression and Bland-Altman plots. (a) Regression analysis for the generalized GPR model and (b) Bland-Altman plot for comparison of the reference walking speed values and the predicted values from the generalized GPR model using the *pca-acc* variable. The upper and lower horizontal lines show the 95% limits of agreement and the middle horizontal line shows the bias.

Table 1 shows the walking speed estimation accuracy of the generalized GPR model for the three different variables: *pca-acc*, *ext-acc*, and *acc*. In this table, the reported mean absolute error (MAE) and root mean square error (RMSE) are used as the measures of accuracy, and the SD shows the precision. Based on this table, when using the *pca-acc* variable, MAE and RMSE are about 5.9±4.7% and 7.9±5.6 cm/s, respectively. Compared to the *ext-acc* and *acc* variables, employing *pca-acc* results in significantly better estimation accuracy. Using analysis of variance (ANOVA), *p*<0.01 shows that the results are statistically significant. Also shown in Table 1 are the walking speed estimation errors when FD features are not being used (the two anthropomorphic features are used in both cases) in the generalized GPR model. As it can be seen, the effect of removing the FD features on the accuracy obtained by the *ext-acc* and *acc* variables is negligible (changes in MAE is <1%), whereas, for *pca-acc*, the accuracy is changed from 5.9% to 15.6%. This shows that the variations in the frequency spectrum of the *pca-acc* variable (which in turn is correlated to the changes in frequency and amplitude of arm swing) is highly correlated to walking speed. The regression analysis and Bland-Altman plots for the predicted walking speed values based on the GPR generalized model using the *pca-acc* variable (and combined FD and TD features) are shown in Fig 5a and 5b, respectively. The black line in Fig 5a shows the line of best fit (*y* = 0.903x+11.7) and the gray line shows the ideal line (*y* = *x*) representing the perfect correlation between the reference and GPR model predicted walking speed. Although the line of best fit slightly deviates from the ideal line due to the prediction errors, the analysis shows a very strong linear correlation between the predicted and reference walking speed values (Pearson’s *r* = 0.9742, *p*<0.001). The Bland-Altman plot shows that, except for a few outliers (mainly at the lower speed range), the error is mainly kept within 95% limits of agreement and that there is no significant systematic dependence of the estimation error on the walking speed. The obtained accuracy using the *pca-acc* variable is better than the MAE of 6.96% in [20] using ceiling-mounted RF transceivers and the RMSE of 8 to 15 cm/s in [21] using wall-mounted RF transceivers for longitudinal in-home walking speed monitoring is used for the early detection of MCI. The positive results herein demonstrate the potential of the proposed wrist-worn method for the monitoring of walking speed as an early marker of health issues. Compared to the systems based on the ceiling and wall-mounted sensors in [20, 21], which are only applicable to confined hallways in single resident homes, the proposed system offers the advantage of being self-contained and can be easily used indoor/outdoor environments.

**Table 1:** Walking speed estimation error (different variables).

Variable	Combined FD and TD features				TD features only			
	MAE (%)	SD (%)	RMSE (cm/s)	SD (cm/s)	MAE (%)	SD (%)	RMSE (cm/s)	SD (cm/s)
***pca-acc***	5.9	4.7	7.9	5.6	15.6	7.0	17.3	7.5
***ext-acc***	12.13	6.97	14.57	7.78	11.8	6.98	14.5	8.28
***acc***	14.4	8.26	16.53	8.49	14.24	6.89	14.3	7.66

Evaluation of the walking speed estimation error based on the generalized GPR model for various variables and feature sets.

As the best walking speed estimation accuracy for the GPR model is obtained using the *pca-acc* variable and a combination of FD and TD features, the reported results in the following sections are based on the same variables and feature sets.

### Comparison Between the Generalized GPR and Subject-Specific GPR Models

Table 2 shows the walking speed estimation errors for the generalized and subject-specific GPR models in various walking speed regimes: slow (50–100 cm/s), normal (100–150 cm/s), and fast (150–200 cm/s). Based on this table, the generalized and subject-specific models have very similar performances for normal and fast walking speed regimes. However, for slow walking regime, the RMSE of the generalized model is about 7.5 cm/s (MAE of 8.9%), whereas the one for the subject-specific model is about 2.6 cm/s (MAE of 2.6%). This can be explained as follows: the generalized model has to fit the model simultaneously across subjects and within each subject. Compared to normal and fast walking, both the frequency and amplitude of arm swing is very weak in slow walking, and for most subjects, the arm swing differs the most at their lowest walking speeds. Intuitively, given that the generalized model has to trade off between overall accuracy and subject-specific accuracy, the model is optimized over the most similar input points, which correspond to arm swing motion in normal and fast walking regimes. To shed further light on this issue, a separate generalized model is trained for the slow walking regime by excluding the data points that correspond to the velocities of above 100 cm/s. The results show that this new model can reduce the RMSE to 5.1 cm/s (and the MAE to 6.2%). This observation suggests that, if one is interested in estimating slow walking speeds (e.g. tracking walking speed of the elderly), a model that is trained specifically for the speed regime of interest may provide a better accuracy.

**Table 2:** Walking speed estimation error (different models).

Speed regime	Generalized model				Subject-specific model			
	MAE (%)	SD (%)	RMSE (cm/s)	SD (cm/s)	MAE (%)	SD (%)	RMSE (cm/s)	SD (cm/s)
**Slow**	8.9	5.6	7.5	4.2	2.6	3.3	2.6	2.6
**Normal**	4.4	4.3	6.6	5.3	4.5	5.5	7.3	7.0
**Fast**	4.5	4.1	9.5	7.3	4.5	3.2	8.1	5.5

Comparison between generalized GPR and subject-specific GPR models in walking speed estimation for various speed regimes: slow (50–100 cm/s), normal (100–150 cm/s), and fast (150–200 cm/s).

### Performance of the Generalized LSR-Lasso Model

**Fig 6:**
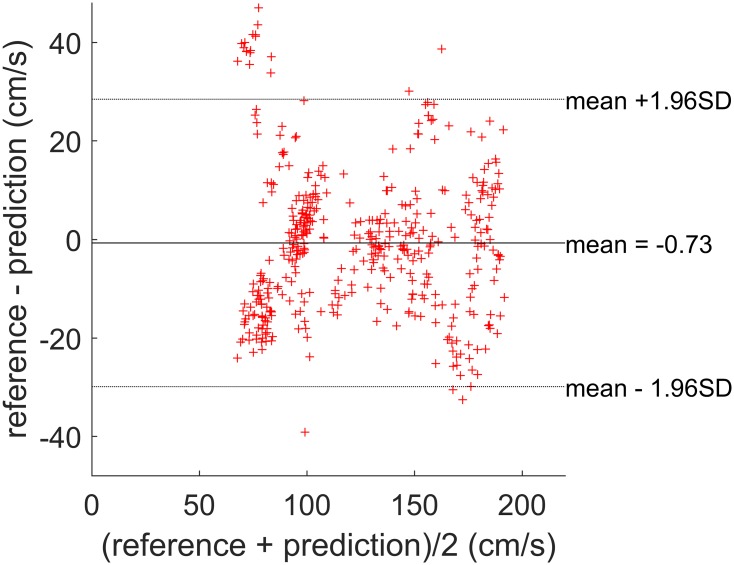
Bland-Altman plot. Comparison of the reference walking speed values and the predicted values based on the generalized LSR-Lasso model.

Fig 6 shows the Bland-Altman plots for the predicted walking speed values based on the generalized LSR-Lasso model. Similar to the generalized GPR model, outliers are mainly in the lower speed range and the error is mainly kept within 95% limits of agreement. The RMSE of the estimated walking speed is about 10.7 cm/s (SD = 7 cm/s) and the MAE is 12.78% (SD = 7.7%). Compared to the generalized GPR model, the LSR-Lasso model has a larger prediction error (*p*<0.01). This comparison shows that the data-driven estimation of the model structure in the GPR model can more effectively capture the influence of each input feature on the output walking speed. A better performance of the GPR model compared to the LSR model has also been observed in [38], where the generalized GPR and LSR models are used to estimate swimming velocity using a waist-worn IMU. In general, once the model is learned, the computational complexity of a nonparametric approach such as GPR for a new estimation depends on the number of training data points (*N*) and is of order *O*(*N*^3^); whereas the one for the parametric approaches such as LSR-Lasso depends on the dimension of the input data space (*d*) and is of order *O*(*d*^3^) [40]. The computational cost can be an important factor when implementing these algorithms in resource-constrained platforms such as wearable devices.

### Testing of the Generalized GPR Model on Outdoor Free Walking Data

**Fig 7:**
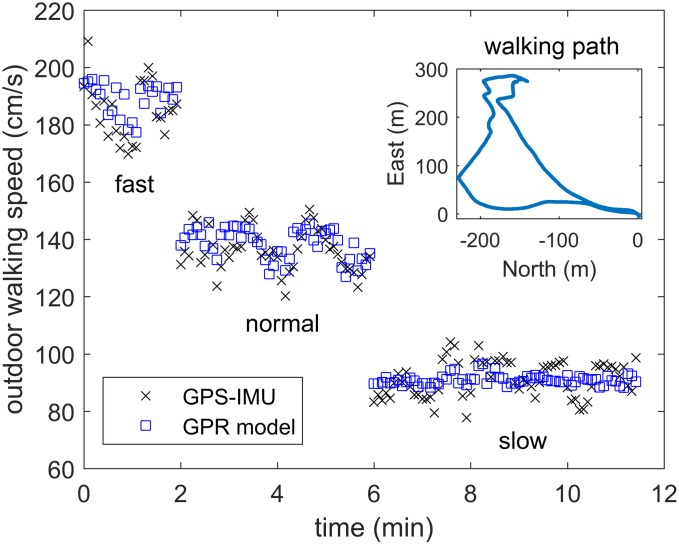
Outdoor walking speed. Estimated walking speed based on the generalized GPR model and the one from GPS-IMU fusion during a 12-min outdoor walking trial.

In this part, the experimental data from the five outdoor free walking trials are used to examine how well the trained generalized GPR model (based on short indoor walking trials that are limited to straight-line walking) will perform for wrist-based walking speed estimation in real-world environment (where the walking path is not limited to a straight line). Fig 7 shows the estimated outdoor walking speed along with the reference speed from our previously verified GPS-IMU fusion algorithm [31], which is more accurate than using GPS alone, for slow, normal, and fast walking speed regimes during a sample 12-min trial from one subject. It can be seen that the generalized GPR model can clearly differentiate between the three walking speed regimes. The variations of walking speed within each speed regime are expected considering the various turns in the walking path (Fig 7, inset) and the natural variations in free walking speed over time. Comparing the predicted walking speed from the five outdoor trials to the GPS walking speed shows a high correlation between the two measurements (Pearson’s *r* = 0.916, *p*<0.001).

## Limitations of the Results

The experimental results presented in this paper may have some limitations. The first part of the limitations is with regard to the measurement errors of the reference system. For the indoor experiments, although the accuracy of the stopwatch is 0.01 s, the human response time for pressing the stopwatch button is in the order of 0.1 s. Additionally, the proposed method is based on the assumption of free arm swing during walking. In reality, this assumption would not be satisfied in occasions such as carrying a bag, putting hand in the pocket, and walking with walkers. However, although there is no arm swing in these situations, because the arm is now fixed to the trunk, the acceleration profile of the wrist will be similar to that of the trunk. Regression model-based walking speed estimation using trunk acceleration is already addressed in the literature [13, 36]. Thus, in real-world applications, these situations can be identified using a proper activity classification algorithm and a separate regression model should be trained for walking speed estimation in such cases.

## Conclusion

A regression model-based human walking speed estimation algorithm is presented, which uses the inertial data from a wrist-worn IMU. The arm swing motion is represented by a novel variable called *pca-acc*, which is highly correlated to walking speed in terms of both temporal and frequency characteristics. Experimental results from 15 young subjects showed that using the proposed *pca-acc* variable will result in significantly better walking speed estimation accuracy compared to the use of raw acceleration variables (*p*<0.01). Using combined TD and FD features of the *pca-acc* variable, a generalized GPR model resulted in accuracy and precision of about 5.9% and 4.7%, respectively. Based on the experimental results, the generalized and subject-specific GPR models tend to perform similarly, except for the slow walking regime (speed <100 cm/s) where a subject-specific model provided better estimation accuracy. Compared to the generalized LSR-Lasso, the generalized GPR model performed significantly better (*p*<0.01) for wrist-based walking speed estimation. Experimental results from a 12-min outdoor walking trial demonstrated the feasibility of using the proposed method for wrist-based walking speed estimation in a real-world environment. In the future, by undertaking a larger study and collecting data across a range of anthropomorphic parameters such as height, weight, and BMI, the generalizability of the proposed method will be further evaluated. Also, the authors plan to carry out clinical investigations to collect real-world data from elderly subjects with diverse ranges of age, weight, and height and to fine tune the regression model for longitudinal walking speed estimation in older adults.
